# Supplementary material for: The interactome of intact mitochondria by cross-linking mass spectrometry provides evidence for coexisting respiratory supercomplexes
Source: Mol Cell Proteomics. 2017 Dec 8;17(2):216–32. doi: 10.1074/mcp.RA117.000470 (PMC5795388; doi:10.1074/mcp.RA117.000470)
Supplement: Supplemental Data [file supp_RA117.000470_133922_0_supp_23978_fzffwf.zip › spectra_annotation/mito_DR_spectra_annotation/23-1-7-1-19-1.pdf]

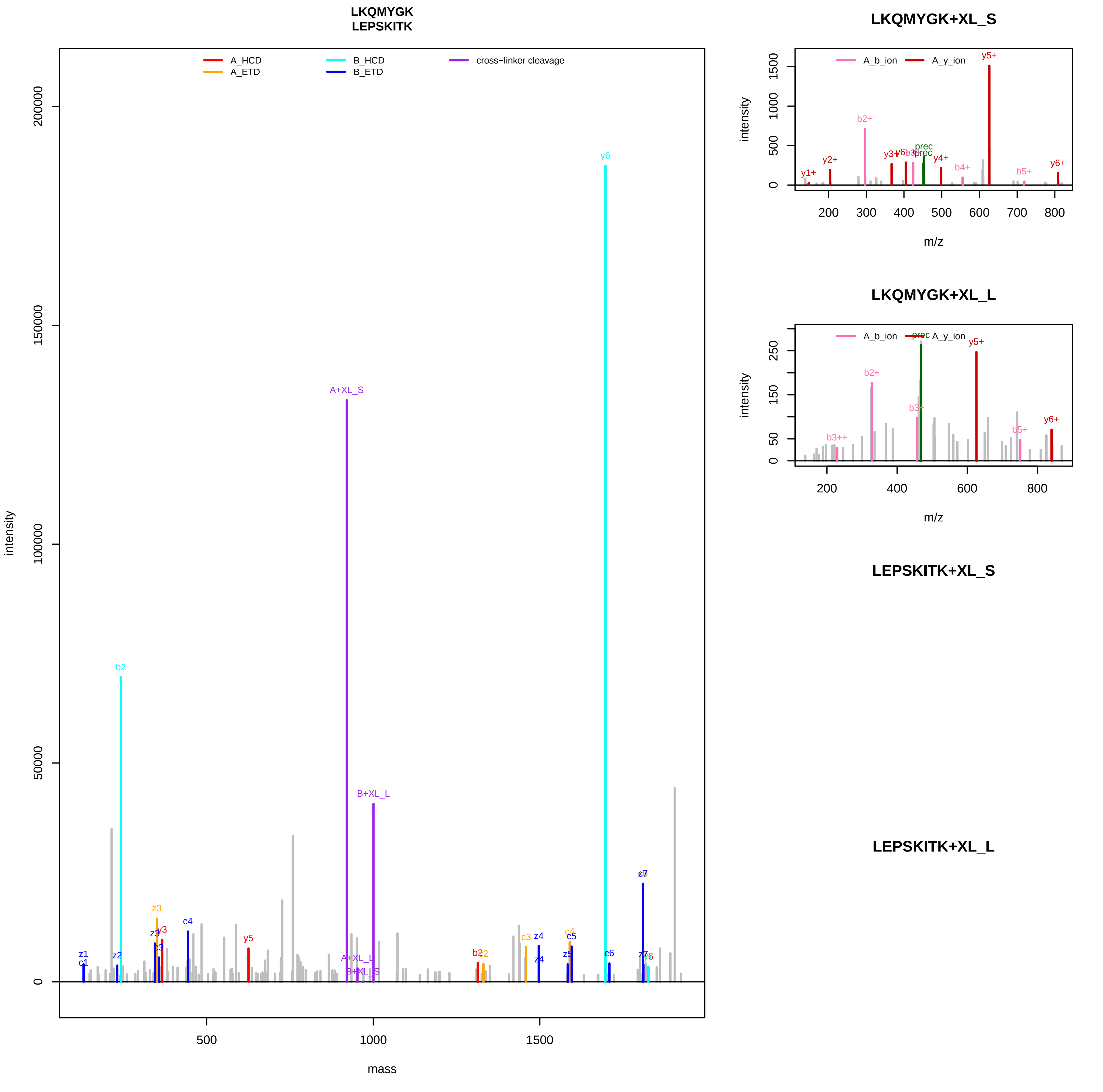

LKQMYGK
LEPSKITK
A_HCD
A_ETD
B_HCD
B_ETD
cross−linker cleavage
intensity
mass
y6
A+XL_S
B+XL_L
b2
z7
c7
z3
c3
y3
c4
y5
c1
z1
z2
A+XL_L
B+XL_S
b2
z2
c3
z4
c5
z5
c4
c6
y6
LKQMYGK+XL_S
A_b_ion
A_y_ion
y5+
b2+
prec
y1+
y2+
y3+
y4+
b4+
b5+
y6+
m/z
LKQMYGK+XL_L
b3++
b3+
LEPSKITK+XL_S
LEPSKITK+XL_L
